# 

**Published:** 1838-04

**Authors:** 


### CONTENTS OF No. X.

OF THE

### British and Foreign Medical Review.

APRIL, 1838.

---

### Part First.—Analytical and Critical Reviews.

PAGE

Art. I.—A History of the Inductive Sciences. By the Rev. W. Whewell, m.a, Fellow and Tutor of Trinity College, Cambridge, &c. . . 317

Art. II.—Dissertatio de Methodo Endermatico. Auctore A. Ahrensen, m.d. . 343

A Dissertation on the Endermic Method. By Dr. Ahrensen.

Art. III.—Elements of Medicine. Vol. I. On Morbid Poisons. By Robert Williams, m.d., Senior Physician of St. Thomas's Hospital . . 353

Art. IV.—1. De Suicidio, Observationibus anatomico-pathologicis illustrato. Scripsit D. J. A. Arntzenius, Med. et Chir. Doct. . . . 368
Of Suicide, illustrated by anatomico-pathological Observations. By D. J. A. Arntzenius, m.d.
2. Das Heimweh und der Selbstmord. Von J. H. G. Schlegel, m.d. &c. . ib.
Nostalgia and Suicide. By Dr. Schlegel.

Art. V.—1. The Philosophy of Health; or, an Exposition of the Physical and Mental Constitution of Man, with a View to the Promotion of Human Longevity and Happiness. By Southwood Smith, m.d., Physician to the London Fever Hospital, &c. Volume Second . . . 380
2. The Philosophy of Living. By Herbert Mayo, f.r.s., Senior Surgeon of the Middlesex Hospital . . . . . . . ib.
3. Management of the Organs of Digestion, in Health and Disease. By Herbert Mayo, f.r.s. &c. . . . . . . . ib.
4. The Economy of Health; or, the Stream of Human Life from the Cradle to the Grave: with Reflections, moral, physical, and philosophical, on the successive Phases of Human Existence, the Maladies to which they are subject, and the Dangers that may be averted. By James Johnson, m.d. . . ib.
5. A Popular Treatise on Diet and Regimen; intended as a Text-book for the Invalid and the Dyspeptic. By W. H. Robertson, m.d. . . ib.
6. A Treatise on Diet; with a View to establish, on practical Grounds, a System of Rules for the Prevention and Cure of the Diseases incident to a disordered State of the Digestive Functions. By J. A. Paris, m.d. &c. Fifth Edition ib.
7. Observations on the Preservation of Health, in Infancy, Youth, Manhood, and Age; with the best Means of improving the moral and physical Condition of Man. By John Harrison Curtis, Esq. . . . . . ib.
8. Education Physique des Jeunes Filles; ou, Hygiène de la Femme avant le Mariage. Par A. M. Bureaud-Riofrey, Docteur en Médecine, &c. . ib.
Physical Education of Young Women, &c. By A. M. Bureaud-Riofrey, m.d. of the Faculty of Paris, &c.
9. The Philosophy of Living; or, the Way to enjoy Life and its Comforts. By Caleb Ticknor, a.m. m.d. . . . . . . 381

Art. VI.—Surgical Observations on Tumours; with Cases and Operations. By John C. Warren, m.d., Professor of Anatomy and Surgery in Harvard University, and Surgeon of the Massachusetts General Hospital . 414

Art. VII.—1. A Treatise on the Diagnosis and Treatment of Diseases of the Chest. Part. I. Diseases of the Lung and Windpipe. By Wm Stokes, m.d. 423
2. Notes et Additions au Traité de l'Auscultation Médiate de Laennec. Par M. Meriadec Laennec, d.m.p. &c., et M. Andral, Professeur à la Faculté de Médecine de Paris, Médecin de l'Hôpital de la Charité, &c. . . ib.
Notes and Additions to the Treatise on Mediate Auscultation of Laennec. By M. Meriadec Laennec, m.d. Paris, &c., and M. Andral, &c.

PAGE
3. Observations on the Surgical Pathology of the Larynx and Trachea, &c. By Wm. Henry Porter, a.m., Vice-President and Professor of the Theory and Practice of Surgery in the Royal College of Surgeons in Ireland, &c. . 423
4. A Treatise on the Diseases and Injuries of the Larynx and Trachea, &c. By Frederick Ryland, Surgeon to the Town Infirmary, Birmingham . 424
5. Traité pratique de la Phthisie laryngée de la Laryngite chronique, et des Maladies de la Voix. Par MM. A. Trousseau et H. Belloc. Ouvrage couronné par l'Académie Royale de Médecine . . . . . ib.
Practical Treatise on Laryngeal Phthisis, Chronic Laryngitis, and Affections of the Voice. By Messrs. A. Trousseau and Belloc.

Art. VIII.—The Philosophy of Marriage, in its social, moral, and physical Relations: with an Account of the Diseases of the Genito-urinary Organs which impair or destroy the Reproductive Function, and induce a variety of Complaints; with the Physiology of Generation in the Vegetable and Animal Kingdoms. By Michael Ryan, m.d &c. . . . . 442

Art. IX.—Hygiène Publique; ou, Mémoires sur les Questions les plus importantes de la Hygiène appliquée aux Professions et aux Travaux d'Utilité publique. Par A. J. B. Parent-Duchatelet, &c. . . . . 447
Public Hygiène; or, Memoirs on the most important Questions of Hygiène applied to Professions and to Works of Public Utility. By A. J. B. Parent-Duchatelet, &c.

Art. X.—Practical Surgery. With one hundred and twenty Engravings on Wood. By Robert Liston, Surgeon . . . . . 457

Art. XI.—Die Pocken-krankheit mit besonderer Rücksicht auf die Pathologische Anatomie. (Es giebt Pocken auf inneren Theilen.) Von Alexander Petzholdt, &c. . . . . . . 469
Small-pox considered with particular Reference to Morbid Anatomy, and more especially to the occurrence of Pocks on internal Parts. By Dr. Petzholdt.

Art. XII.—On Diseases of the Rectum. By James Syme, f.r.s.e. . . 480

Art. XIII.—1. Memoirs on the Nervous System. By M. Hall, m.d. f.r.s. l. & e. 486
2. Observations on the Structure and Functions of the Spinal Cord. By R. D. Grainger, Lecturer on Anatomy and Physiology . . . ib.
3. Powers of the Roots of the Nerves, in Health and in Disease; likewise on Magnetic Sleep. By Herbert Mayo, f.r.s. &c. . . . ib.

Art. XIV.—Changes produced in the Nervous System by Civilization, considered according to the Evidence of Physiology and the Philosophy of History. By Robert Verity, m.d. &c. . . . . . 540

---

### Part Second.—Bibliographical Notices.

Art. I.—Researches into the Physical History of Mankind. By James Cowles Prichard, m.d. f.r.s. &c. Third Edition. Vol. II.; containing Researches into the Physical Ethnography of the African Races . . . 543

Art. II.—A concise Treatise on Operative Surgery. By W. P. Cocks, Surgeon . 544

Art. III.—The Question concerning the Sensibility, Intelligence, and Instinctive Actions of Insects. By David Badham, m.d. &c. . . . 545

Art. IV.—On Warming and Ventilating; with Directions for making and using the Thermometer-Stove, or Self-regulating Fire, &c. By Neill Arnott, m.d. 546

Art. V.—A Treatise on Ruptures. By Wm. Lawrence, f.r.s. &c. . . 547

Art. VI.—The Connexion of Natural and Divine Truth; or, the Study of the Inductive Philosophy considered as subservient to Theology. By the Rev. B. Powell 548

Art. VII.—Anatomical, Pathological, and Therapeutic Researches upon the Disease known under the Name of Gastro-Enterite, &c. By P. C. A. Louis, m.d. Translated from the French, by H. J. Bowditch, m.d. . . 549

Art. VIII.—De Physiologia Tenotomiæ Experimentis illustrata. By Prof. Ammon 550

On the Physiology of the Division of Tendons in Surgical Operations, illustrated by Experiments. By F. A. von Ammon, m.d.

### PART THIRD.—Selections from Foreign Journals.

### ANATOMY AND PHYSIOLOGY.

PAGE

On the Structure of the Iris in Birds, and the Mechanism by which it is moved. By Dr. AUGUST KROHN . . . . . 553

Anatomical Observations and Remarks. By Dr. KRAUSE . . . 554

Nature of the Degeneration of the Kidney in Bright's Disease. By Dr. GLUGE . 556

Absence of the Right Lung—(Cyanosis) . . . . ib.

Existence of the Polystoma Sanguicola in the Human Blood. By Dr. DELLE CHIAJE ib.

### PATHOLOGY, PRACTICAL MEDICINE, AND THERAPEUTICS.

Notice of the Influenza which prevailed at Lisbon during the Month of February, 1837. By Dr. LIMA LEITAO . . . . . 557

The Influenza in Copenhagen in the Winter of 1836-7. By Dr. OTTO . 558

Method of treating Intermittent Fevers in the Infirmary of Clinical Medicine of the Surgical School of Lisbon. By Dr. LIMA LEITAO . . . ib.

Observations on the Plague. By Dr. BULARD . . . . 560

On the Medicinal Powers of the Caïnca. By Dr. ROBREDO . . . 562

Notice of the Bafureira of Cape de Verd. By Dr. B. A. GOMES . . 563

Febrifuge Properties of the Chloride of the Oxide of Sodium. By Dr. GOUZÉE . ib.

On Obliteration of the large Arteries. By M. LEGROUX . . . 564

Particular Kind of Swelling of the Tonsils, Uvula, and soft Palate. By Dr. RÖSCH ib.

Chronic Hydrocephalus, cured by accidental Paracentesis. By Dr. HÖFLING . 565

Treatment of Diseases with Milk and Whey. By Dr. LEVISEUR . . ib.

On [supposed] Inflammation of the Pancreas, observed during an Epidemic Parotitis. By Dr. CONTI . . . . . . 567

New Method for the Cure of Stammering. By Dr. VOISIN . . . 568

### SURGERY.

On the Treatment of Aneurism by Ice. By M. REYNAUD . . . 568

On Dislocations of the Shoulder-joint. By M. VELPEAU . . . 569

On sudden Dilatation, a new Method employed by Professor LALLEMAND in destroying Strictures of the Urethra . . . . 572

On a new Method of Treating the Hectic Fever which follows the Operation for Empyema. By M. FUSTER . . . . . 573

Fracture of the Ribs by Contre-coup. By Dr. MONDIERE . . . 574

Treatment of Wounds by the Affusion of Cold Water. By M. GODIN . . ib.

Method of Promoting a Flow of Blood after Venesection. By Dr. BURDACH . ib.

On the use of Soot in Diseases of the Bladder. By M. ANDRÉ GIBRIN . 575

On the use of Chloride of Lime in Wounds attended with much Pain. By Dr. CHOPIN ib.

Treatment of incised Wounds by the Membranes of the Egg. By Dr. HEUSSNER . 576

### MIDWIFERY.

Report of the Berlin Lying-in Hospital, from October 1, 1829, to December 31, 1835. By Professor BUSCH . . . . . 576

Account of a Case of Ruptured Uterus. By Dr. NAEGELÉ, jun. . . 577

Resuscitation of an Infant after Interment. By Dr. WAGNER . . 582

Observations on the Etiology of the Thrombus, or Bloody Tumour of the Head of the Fœtus in tedious Labours. By Dr. CARVALHO . . . 584

### PART FOURTH.—Selections from British Journals.

### ANATOMY AND PHYSIOLOGY.

PAGE

History of a Female who has four Mammæ and Nipples. By ROBERT LEE, M.D. 385

Remarks on the Fallacy of Professor TIEDEMANN's Comparison of the Negro Brain and Intellect with those of the European. By ANDREW COMBE, M.D. . ib.

Second Report of the London Committee of the British Association for the Advancement of Science, on the Motions and Sounds of the Heart . . 589

On the Cerebral Extremity of the Optic Nerve. By SAMUEL SOLLY, Esq. . 593

### PATHOLOGY, PRACTICAL MEDICINE, AND THERAPEUTICS.

Another Instance of supernumerary Mammæ. By W. H. MOORE, Esq. . 594

On the Frequency of the Presence of the Trichocephalus Dispar in the Human Intestines. By O'B. BELLINGHAM, M.D . . . . . ib.

Enquiry as to whether Laryngismus Stridulus is a Spasmodic or Paralytic Affection. By WILLIAM GRIFFIN, M.D. . . . . . . 595

On the Use of the Stethoscope in the Practice of Midwifery. By Dr. D. C. NAGLE 596

Observations on Animal Magnetism. By Dr. SIGMOND . . . 598

Practical Observations on Delirium cum Tremore. By WM. MUNK, M.D. . 600

On some of the Morbid Conditions of the Valves of the Aorta. By Dr. WATSON . ib.

Cases of aggravated and irregular Forms of Hysteria. By THOMAS LAYCOCK, Esq. 601

Statistical and Pathological Report of the Royal Infirmary of Edinburgh, for the Years 1833, 34, 35, 36, and half of 1837. By JOHN HOME, M.D. . ib.

Observations relative to Changes in the Crystalline Lens, and the Cause and Cure of Cataract. By Sir DAVID BREWSTER, K.H. F.R.S. . . . 602

Case of Pericarditis. By THOMAS WATSON, M.D. . . . . . 604

On the Use of Arsenic in Plethora. By Mr. LAW . . . 605

Iodine and Conium in Tubercular Phthisis Pulmonalis. By Sir C. SCUDAMORE, M.D. 606

On Rupture of the Heart, &c. By JOHN THURNAM, Esq. . . . 607

### SURGERY.

Report of Surgical Cases, in the Glasgow Royal Infirmary. By Dr. DAVIDSON . 607

Singular Case in which a Steel Fork was extracted from the Back. By Dr. BURNES 609

Rupture of an Aneurism of the common Carotid Artery. By Dr. ROBERTSON . 610

Observations on the Pathology of Staphyloma. By T. WHARTON JONES, Esq. . 612

Fatal Convulsion during the Injection of Nævus. By Messrs. PAGET and FULLAGAR 613

### MIDWIFERY.

Spontaneous Amputation of the Limbs in Utero. By RICHARD SMITH, Esq. . 614

---

### PART FIFTH.—Medical Intelligence.

Experiments and Observations on Hanging. By Dr. CASPER . . 615

The Controversy between Drs. GRANT and HALL, and Mr. NEWPORT, respecting Discoveries in the Nervous System . . . . 620

"The Rival Discoverers" . . . . . . 621

Complete Anticipation of Dr. Marshall Hall's Doctrine of "the Reflex Function," by Prochaska . . . . . . 623

The Cholera in Naples . . . . . . 625

Royal Medical Appointments for Scotland . . . . 626

OBITUARY—HENRY EARLE, F.R.S., Surgeon Extraordinary to the Queen . 627

JOHN HOME, M.D. . . . . . 628

BOOKS RECEIVED FOR REVIEW . . . . . ib.

